# A Master Switch? *estrp* Directs Blood Vessel Development in Zebrafish

**DOI:** 10.1371/journal.pbio.0040017

**Published:** 2005-12-20

As the primary distributor of nutrients and oxygen to cells throughout the vertebrate body, the circulatory system provides life support to the body's tissues and organs. Consequently, the cellular and developmental signals that control development of this critical organ system, composed of the heart, the blood vessels, and the blood cells they contain, are the subject of intense study. Medical researchers also find it valuable to know the signals that control blood vessel development because solid tumor masses depend on infiltrating blood vessels to grow.

Circulatory system development proceeds similarly in all vertebrates, with the heart and aorta beginning to form early on (about the point at which recognizable structures like the head begin to form). Specialized cells called angioblasts create blood vessels that bud off from the aorta and branch out to form the rest of the animal's vasculature. With its transparent embryos that develop outside the mother, the zebrafish is a favored model organism for circulatory system development.

Zebrafish sporting a mutation called *cloche* (a French word referencing the mutant animal's bell-shaped heart) lack both blood vessels and blood cells. This discovery produced an important insight: the loss of two different cell types with one mutation suggests that both types of cells may arise from a common precursor at some point in early development. But until now, no single protein has been identified that differentiates the hemocytes that are destined to become blood cells from the angioblasts destined to become blood vessels. In a new paper, Saulius Sumanas and Shuo Lin describe a gene called *etsrp* that is specifically required for blood vessel development.

The authors had originally discovered *etsrp* in a screen they conducted to find genes whose expression is altered by the *cloche* mutation. To learn what exactly *etsrp* might be doing during development, the authors first looked for clues in its sequence by comparing it to the sequence of similar known proteins, the Ets family of transcription factors. The chromosomal location of *etsrp* suggested it had arisen as a result of a genetic duplication of the founding member of this family, the *ets1* gene (thus its name, which stands for “Ets1-related protein”). It's been known for some time that Ets family transcription factors play many roles in the development of the circulatory system by binding DNA and controlling the expression of genes critical to the development of circulatory system cell lineages, suggesting that *etsrp* may also be involved.

With this information in hand, the authors set out to investigate where and when *etsrp* is expressed during zebrafish development by looking for the presence of *etsrp* mRNA in developing embryos. They found *etsrp* mRNA expressed early in development in tissues that eventually give rise to blood vessels; later on, they saw *etsrp* mRNA expressed in more mature blood vessel structures throughout the animal. These findings suggested that *etsrp* might be involved in the designation of these structures during development. To see whether *etsrp* is required for blood vessel development, Sumanas and Lin disrupted the expression of *etsrp* in developing embryos. Interestingly, loss of *etsrp* resulted in the absence of blood vessels in the developing animal, even though the animals were able to make blood cells normally; the embryos' blood cells remained clumped at their formation site next to the yolk extension, rather than entering circulation. It appeared there just weren't any blood vessels around for the blood cells to move through. In support of this conclusion, Sumanas and Lin could not detect evidence that any body cells expressed the surface proteins normally associated with blood vessel identity. Therefore, expression of these markers appears to be dependent on the presence of *etsrp*. [Fig pbio-0040017-g001]

**Figure pbio-0040017-g001:**
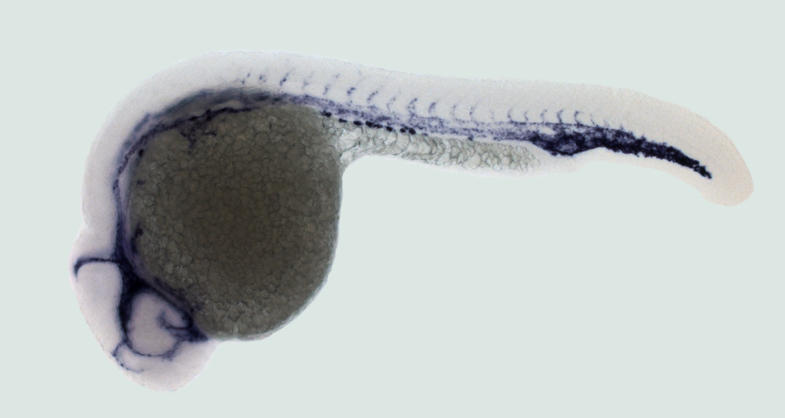
The transparent zebrafish embryo helped researchers identify a putative master gene controlling blood vessel development

If the expression of blood vessel–specific markers is dependent on *etsrp*, is it also true that *etsrp* is sufficient for expression of these markers? The authors found that artificially causing the overexpression of *etsrp* in early embryos resulted in the inappropriate expression of blood vessel–associated proteins in cells that normally do not express them. Finally, since *cloche* mutants also lack expression of blood vessel–specific markers (in addition to lacking blood cell–specific markers), the authors wondered whether artificially expressing *etsrp* in *cloche* mutants could restore the expression of markers associated with the development of blood vessels. Indeed, they found that *etsrp* could restore the expression of the blood vessel–specific marker, *flk1*, in *cloche* mutant embryos. Taken together, these data indicate that *etsrp* is both necessary and sufficient for blood vessel development in the zebrafish, lending important insights into our understanding of circulatory system development. *—Caitlin Sedwick*

